# Investigations on the Properties and Performance of Mixed-Matrix Polyethersulfone Membranes Modified with Halloysite Nanotubes

**DOI:** 10.3390/polym11040671

**Published:** 2019-04-11

**Authors:** Sylwia Mozia, Amanda Grylewicz, Michał Zgrzebnicki, Dominika Darowna, Adam Czyżewski

**Affiliations:** Faculty of Chemical Technology and Engineering, Institute of Inorganic Chemical Technology and Environment Engineering, West Pomeranian University of Technology, Pułaskiego 10, 70-322 Szczecin, Poland; amanda.grylewicz@zut.edu.pl (A.G.); michal.zgrzebnicki@zut.edu.pl (M.Z.); dominika.darowna@zut.edu.pl (D.D.); adam.czyzewski@zut.edu.pl (A.C.)

**Keywords:** polyethersulfone, membrane, halloysite nanotubes, ultrafiltration, fouling

## Abstract

Ultrafiltration (UF) polyethersulfone (PES) membranes were prepared by wet phase inversion method. Commercial halloysite nanotubes (HNTs) in the amount of 0.5–4 wt % vs PES (15 wt %) were introduced into the casting solution containing the polymer and *N*,*N*-dimethylformamide as a solvent. The morphology, physicochemical properties and performance of the membranes were characterized by scanning electron microscopy (SEM) and atomic force microscopy (AFM), zeta potential, porosity and contact angle analyses, as well as permeability measurements. Moreover, the antifouling properties of the membranes were evaluated during UF of a model solution of bovine serum albumin (BSA). The research revealed a positive influence of modification with HNTs on hydrophilicity, water permeability and antifouling properties of the PES membranes. The most significant improvement of permeability was obtained in case of the membrane containing 2 wt % of HNTs, whereas the highest fouling resistance was observed for 0.5 wt % HNTs content. It was found that a good dispersion of HNTs can be obtained only at loadings below 2 wt %. Based on the results a relation between severity of membrane fouling and surface roughness was proved. Moreover, an increase of the roughness of the modified membranes was found to be accompanied by an increase of isoelectric point values.

## 1. Introduction

Polyethersulfone (PES) is widely used for preparation of ultrafiltration (UF) and microfiltration (MF) membranes applied for water and wastewater treatment [1,2]. However, the main drawback of its application in this field is relatively low hydrophilicity which contributes to the susceptibility of the membranes to fouling [3]. Fouling is one of the major drawbacks in membrane separation processes. The particles, colloids or dissolved organics present in feed solution can be adsorbed/deposited on the membrane surface and within its pores leading to permeate flux decline or even membrane damage [4,5,6,7]. One of attempts proposed to reduce the fouling problem is introduction of various fillers into the polymer membrane structure. The development of nanotechnology has created a new path in mixed-matrix membrane development based on application of various nanomaterials. In case of PES membranes the nanofillers such as pure or modified TiO_2_ [8], SiO_2_ [9], Al_2_O_3_, halloysite nanotubes (HNTs) [10] and carbon nanotubes (CNTs), as well as silver [11] or copper nanoparticles (NPs) [12] have been proposed for the modification purpose. Application of these nanomaterials was reported to improve antifouling performance via increase of membrane hydrophilicity [8,9,13,14].

Halloysite nanotubes are a kind of aluminosilicate clay, with the Si/Al ratio of 1:1 and a general formula of Al_2_Si_2_O_5_(OH)_4_·nH_2_O [15]. They are natural hollow tubular nanomaterials with a 1D structure characterized by chemically active external and internal surfaces [16,17]. HNTs have large surface area and porous microstructure which make them a good adsorbent of dyes and heavy metals [18]. Moreover, HNTs possess hydrophilic groups on the surface, which can increase the hydrophilicity of the membrane [11].

The number of literature reports on application of neat halloysite nanotubes for membranes preparation is low. The unmodified HNTs were applied for preparation of polymeric membranes made of PES [10], polyamide (PA) [19,20], polysulfone (PSU) [21], polystyrene (PS) [22], chitosan [23], polyacrylonitrile (PAN) [24] and poly(vinyl chloride) (PVC) [25]. These membranes were applied in MF [22], UF [10,22,25] and reverse osmosis (RO) [19,20] processes.

Thin film nanocomposite (TFC) reverse osmosis membranes modified with halloysite nanotubes were obtained by incorporating different amount of the nanomaterial into the polyamide selective layer via in situ interfacial polymerization [19,20]. Introduction of HNTs (0.01–0.1 wt/vol%) resulted in an improvement of hydrophilicity, surface roughness and water flux of the membranes and the effect was more visible at higher content (0.1 wt/vol%) of the nanotubes. The increase of pure water flux (PWF) was ascribed to higher hydrophilicity of the membranes and tubular structure of HNTs. Separation performance determined with reference to NaCl rejection slightly decreased after addition of HNTs, whereas antifouling properties evaluated on a basis of permeate flux during bovine serum albumin (BSA) filtration were significantly improved. That was attributed to the more negative surface charge of the membranes upon addition of HNTs [19,20]. To improve the dispersion of HNTs in polysulfone membranes, application of polyetheramine was proposed [21]. An increase of the hydrophilicity of the membranes was observed and, additionally, an enhancement of water uptake in case of membranes containing higher concentration (7–10 wt %) of HNTs was reported. Furthermore, a decrease of porosity of the membranes with filler loading up to 5 wt % was found, whereas a higher loading of the nanomaterial led to the porosity increase. However, the authors did not present any data on permeability or separation properties of the membranes [21]. Halloysite nanotubes were also proposed as a modifying agent of polystyrene membranes [22]. The effect of solvent type (tetrahydrofuran (THF) vs *N*-methyl-2-pyrrolidone (NMP)) on the physical, mechanical and thermal properties of the membranes was evaluated. The application of THF led to the formation of a microporous membrane dedicated for MF, whereas in the presence of NMP a nanoporous UF membrane was obtained. In case of both membranes the increase in the filler content resulted in an increase of water flux and improvement of rejection properties [22]. An improvement of PWF was also reported for PES ultrafiltration membranes modified with pristine HNTs (1–3 wt % vs polymer), prepared using *N*,*N*-dimethylacetamide (DMAc) as a solvent with addition of polyvinylpyrrolidone (PVP) and acetone [10].

Nonetheless, most of literature reports on the application of HNTs for preparation of polymeric membranes refer to the nanotubes modified with Ag [11], Ag-ZnO [26], silver nanoparticles-reduced graphene oxide (AgNPs-rGO) [27], Ag-chitosan [11], Cu [12], TiO_2_ [28], [3-(2-Aminoethylamino)propyl]trimethoxysilane (AEAPTMS) [29], dopamine [30], polydopamine [4], poly(sodium4-styrenesulfonate) [31], 3-aminopropyltriethoxysilane [32], 2-methacryloyloxyethyl phosphorylcholine (MPC) [33], dextrane [34], N-halamine [35], lysozyme (Mucopeptide N-acetylmuramoylhydrolase) [36] and Fe_3_O_4_ [37]. The modifications with Ag or Cu are aimed at improvement of antibacterial properties of the membranes, while the other approaches are focused mainly on an increase of permeate flux and antifouling performance.

In case of UF membranes made of PES, halloysite nanotubes modified by N-halamine [35], dextrans [34], AgNPs-rGO (AgNPs-HNTs-rGO) [27], MPC [33] and copper ions [12] were applied as modifying agents. Duan et al. [35] observed that PWF in case of a membrane containing 1 wt % of HNTs modified by N-halamine was about three-times higher than that of the unmodified one. However, the best antifouling properties were observed for a membrane containing as much as 3 wt % of NPs [35]. Similarly, application of 1 wt % of AgNPs-HNTs-rGO composite presented by Zhao and others [27] resulted in ca. three-times increase of PWF, while its decrease at higher amount of the filler was found. However, the permeate flux at 3 wt % was still higher (for 34.6%) compared to the unmodified membrane. The introduction of AgNPs-HNTs-rGO had a positive effect on BSA fouling mitigation, similarly to the previously described examples [27]. Comparable results were obtained after the introduction of dextran modified halloysite into PES UF membranes; however, the most significant PWF increase was found for 3 wt % addition of NPs [34]. The application of HNTs modified with copper ions resulted in 17–64% increase of PWF for 1–3 wt % of the NPs, respectively [12]. Unfortunately, the results presented in the above papers were not compared with those obtained using unmodified halloysite. Therefore, the effect of HNTs modification cannot be fully evaluated.

The above review of the present state of the art in the area of halloysite-modified membranes revealed that the reports on the influence of pristine HNTs on the properties of PES UF membranes are very limited. Most of papers refer to application of modified HNTs, while the effect of the neat nanomaterial on the performance of UF membranes is not well described. The main aim of the present study was to examine the effect of pristine halloysite nanotubes on the physicochemical properties and fouling resistance of the modified PES UF membranes. We have applied a simple blending method of production of the mixed-matrix membranes which does not require any post-treatment steps of modification of a membrane surface in order to deposit or fix HNTs.

The PES (15 wt %) and PES/HNTs membranes were prepared via wet phase inversion method using *N*,*N*-dimethylformamide (DMF) as a solvent. Commercial halloysite nanotubes were applied in concentration of 0.5–4 wt % with reference to PES. The membranes were characterized based on scanning electron microscopy (SEM), atomic force microscopy (AFM), zeta potential, porosity, contact angle (CA) and pure water flux measurements. Membrane fouling was evaluated using bovine serum albumin as a model foulant.

## 2. Materials and Methods

Polyethersulfone (Ultrason E6020P) was obtained from BASF SE (Ludwigshafen, Germany). Halloysite nanotubes were supplied by Sigma Aldrich (Saint Louis, MO, USA). *N*,*N*-dimethylformamide (DMF, puriss p.a.) was provided by Avantor Performance Materials Poland S.A. (Gliwice, Poland). Bovine serum albumin (BSA; Probumin) was purchased from Merck KGaA (Darmstadt, Germany). In all experiments pure (deionized) water (type 2, 0.066 µS/cm) from Elix 3 (Millipore, Merck KGaA, Darmstadt, Germany) was applied.

Pure PES membranes and PES/HNTs membranes were prepared by wet phase inversion method. In case of the unmodified membrane the polymer (15 wt %) was dissolved in DMF (85 wt %). The homogeneous casting dope was casted onto glass plate using an automatic film applicator (Elcometer 4340, Elcometer Ltd., Manchester, UK) with the knife gap of 0.1 mm, and subsequently immersed in a pure water bath (20+/−1 °C) to complete the phase inversion process.

The PES/HNTs membranes casting dopes (15 wt % PES) were prepared by mixing a dispersion of HNTs (0.5–4% by weight of the polymer) in a solvent (10 cm^3^) with the previously made solution of PES in DMF (40 cm^3^). The dispersion of HNTs in the solvent was prepared by sonication for 30 min using ultrasonic probe (Vibra-cell VCX-130, Sonics, Newtown, CT, USA; output power 130 W, frequency 20 kHz, amplitude 80%). After addition of the HNTs dispersion to the polymer solution the casting dope was mixed alternately using (i) a magnetic stirrer at temperature of 55–60 °C and (ii) sonication in an ultrasonic bath (Sonic-6D, Polsonic, Warsaw, Poland; output power 320 W, frequency 40 kHz) for 2 h, 15 min by turns. After degassing at room temperature, the membranes were casted using the automatic film applicator as described above.

The morphology of HNTs was analyzed using transmission electron microscope (TEM) FEI Tecnai F20 (FEI Company, Hillsboro, OR, USA) equipped with energy dispersive X-ray (EDX) detector. Phase composition was determined by X-ray diffraction (XRD) using the PANalytical Empyrean diffractometer (PANalytical B.V, The Netherlands) with CuKα (λ = 1.5405980 Å) radiation. The Fourier transform infrared-attenuated total reflection (FTIR-ATR) spectra were collected using Nicolet 380 FT-IR spectrophotometer equipped with an ATR accessory (Smart OrbitTM, Thermo Electron Corp., Waltham, MA, USA). The isoelectric point (pH(I)) of the HNTs was examined using Zetasizer Nano-ZS (Malvern Instruments Ltd., Malvern, UK) equipped with Multi Purpose Titrator MPT-2 and a degasser. The sample was dispersed in ultrapure water and the pH was adjusted using NaOH and HCl solutions.

Porosity of the membranes was determined by gravimetric method. At first, membranes samples with dimensions of 5 × 5 cm were surface dried and weighed on the analytical balance. After subsequent drying in an oven at temperature of 105 °C for 1.5 h the samples were weighed again. Porosity was calculated based on Equation (1):

(1) $$P=\frac{\left(w_{w}-w_{d}\right)/\rho_{H_{2}O}}{\left(\frac{w_{w}-w_{d}}{\rho_{H_{2}O}}\right)+\frac{w_{w}}{\rho_{PES}}}\times 100\%$$

where: *w*_w_—weight of wet membrane, *w*_d_—weight of dry membrane, *ρ*_H20_—density of water at 20 °C (0.9982 g/cm^3^) and *ρ*_PES_—density of polymer (1.37 g/cm^3^).

Static water contact angle (SCA) of the membranes was determined using a goniometer (type 260, ramé-hart instruments co., Succasunna, NJ, USA) by sessile drop method. The volume of the water drop was 10 µL. Additionally, the advancing (ACA) and receding (RCA) contact angles were determined by slowly increasing (from 3 to 11 µL) and subsequently reducing the volume of water drop. The results are mean values of at least 10 separate measurements.

The zeta potential of the membranes was evaluated using SurPASS 3 analyzer (AntonPaar, Graz, Austria). As an electrolyte, a solution of 0.001 KCl in ultrapure water was used. The pH was adjusted using HCl and KOH solutions. The isoelectric point was calculated from at least 2 repeated measurements.

Surface topography of the membranes was analyzed using atomic force microscope (AFM; NanoScope V Multimode 8, Bruker Corp., Billerica, MA, USA) with silicon nitride probe in the ScanAsyst mode. The roughness was given in terms of the mean roughness (R_a_) calculated from the arithmetic average of the values of the surface height deviations measured from the mean plane. The R_a_ was evaluated on a basis of at least five AFM images (10 µm x 10 µm) of the skin layer of the membrane (which was in contact with non-solvent during membrane preparation) using the NanoScope Analysis software. To avoid the shrinkage of the membrane due to the drying at high temperature [38,39,40], the examined membranes were dried in ethanol at room temperature before the AFM analysis.

Morphology of the membranes was analyzed using ultra-high-resolution field-emission scanning electron microscope (UHR FE–SEM) Hitachi SU8020, Krefeld, Germany. Before SEM analysis, a small piece of a membrane, previously dehydrated in ethanol and broken in liquid nitrogen, was sputtered with a chromium layer (Q150T ES Quorum Technologies Ltd., Lewes, UK). The analysis was carried out in two modes: using (i) secondary electrons (SE; accelerating voltage 5 kV) and (ii) backscattered electrons (BSE; accelerating voltage 15 kV).

The pure water flux (PWF) was determined based on ultrafiltration of pure water at transmembrane pressures of TMP = 1, 2 and 3 bar. The membrane (0.0025 m^2^) was mounted in a stainless steel membrane module with a 1.194 mm feed spacer.

Antifouling properties of the membranes were determined at TMP = 2 bar and feed cross flow velocity of 1 m/s. Concentration of BSA was 1 g/dm^3^ (pH = 6.85). The process was carried out for 2 h. The BSA rejection was determined based on concentration of total organic carbon (TOC) in feed (*C_0_*) and in permeate (*C_p_*):

(2) $$R=\frac{C_{0}-C_{p}}{C_{0}}\times 100\%$$

TOC concentration was measured using organic carbon analyzer (multi N/C 3100, Analytik Jena, Germany).

## 3. Results and Discussion

### 3.1. Characterization of HNTs

**Figure 1:**
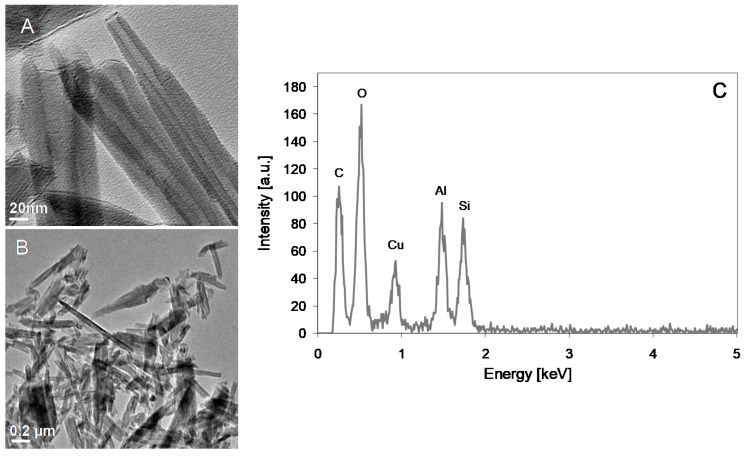
TEM images (**a**,**b**) and EDX pattern (**c**) of halloysite nanotubes.

Figure 1 presents TEM images (Figure 1A,B) and EDX pattern (Figure 1C) of halloysite nanotubes. The length of halloysite nanotubes was in the range of 150–1250 nm, the internal diameter changed from 11 to 28 nm and the wall thickness amounted to 5–23 nm. The EDX analysis of HNTs (Figure 1C) revealed the reflections from aluminum, silicon and oxygen corresponding to the chemical composition of the material (Al_2_Si_2_O_5_(OH)_4_ּ·nH_2_O). The peaks representing carbon and copper are due to the TEM grid used as the sample support.

**Figure 2:**
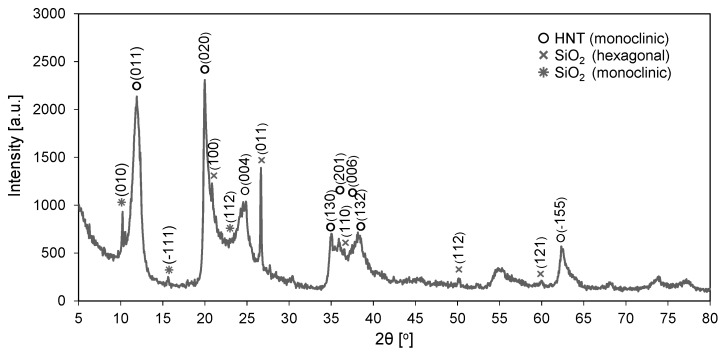
Diffractogram of halloysite nanotubes.

XRD pattern of halloysite nanotubes is shown in Figure 2. The reflections positioned at 2θ angles of 11.6° (011), 20.0° (020), 24.6° (004), 35.0° (130), 35.8° (201), 37.5° (006), 38.2° (132), 62.5° (−155) were attributed to halloysite of monoclinic crystallographic system (ICDD #00-060-1517). The reflection peaks representing SiO_2_ with hexagonal structure can be observed at 20.9° (100), 26.7° (011), 36.6° (110), 50.2° (112), 60.1° (121) (ICDD #01-085-0865), while the reflections corresponding to the monoclinic system are positioned at 2θ of 10.1° (010), 15.8° (−111), 23.1° (112) (ICDD #01-080-5552).

**Figure 3:**
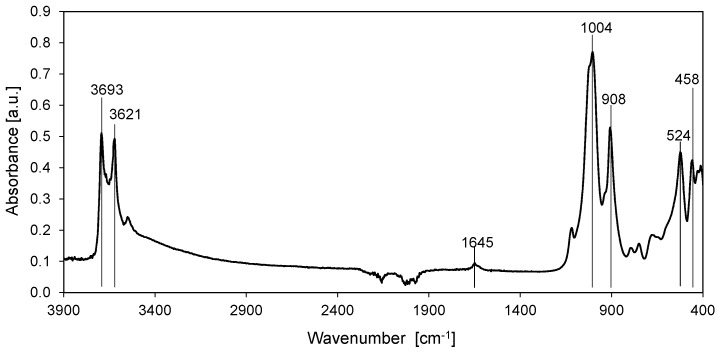
FTIR-ATR spectrum of halloysite nanotubes.

Figure 3 shows the ATR/FTIR spectrum of HNTs. The absorption bands at wavenumbers of 3693 cm^−1^ and 3621 cm^−1^ correspond to the stretching vibrations and at 908 cm^−1^ to the deformation vibrations of –OH groups occurring on the surface and inside of the HNTs. The band at 1650 cm^−1^ represents the deformation vibrations of the adsorbed water. The band at 1004 cm^−1^ can be attributed to the stretching vibrations of Si–O, at 524 cm^−1^ to the deformation vibrations of Al–O–Si and at 458 cm^−1^ to the bending vibrations of Si–O–Si [21,41].

### 3.2. Physicochemical Properties of Membranes

The introduction of HNTs into PES membrane matrix affected its physicochemical properties. A slight increase of the overall porosity from 70% in case of the unmodified membrane to 72% in case of the membranes containing the nanofiller was observed. However, there was no relation between the amount of the introduced modifier and porosity, which may be due to too low concentration of the modifier or too narrow range of HNTs content in the membranes. An increase in the porosity after introduction of halloysite into polyvinylidene fluoride (PVDF) membrane was also observed by Zeng et al. [30]. The authors explained that by (i) the influence of nanoparticles on the kinetic of phase inversion process by accelerating the diffusion rate between solvent and water and (ii) the inhibition of the diffusion process resulting from the increased solid (HNTs) content in the casting dope. The final porous structure was a result of both phenomena [30].

**Figure 4:**
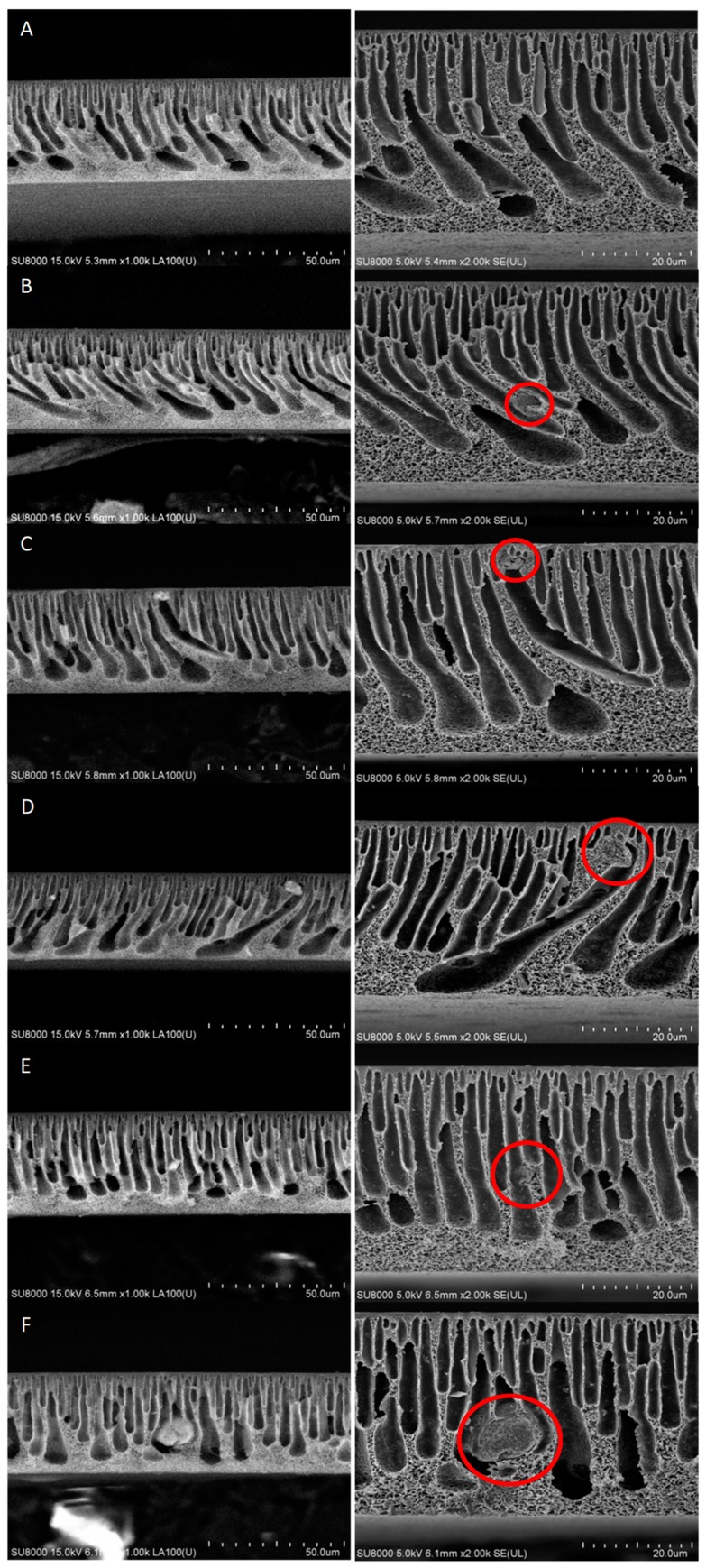
SEM-BSE (left column) and SEM-SE (right column) images of unmodified membrane (**A**) and HNTs-modified membranes (**B**—0.5%HNT, **C**—1%HNT, **D**—2%HNT, **E**—3%HNT, **F**—4%HNT).

SEM images of the membranes cross sections taken using BSE and SE modes are shown in Figure 4. All membranes exhibited an asymmetrical structure with a thin separation layer in the upper part of the membranes. Throughout the cross-section, the oblong, finger-shaped pores tapering towards the skin are visible. Between the finger-like pores and in the bottom part of the membranes a spongy structure can be seen.

**Figure 5:**
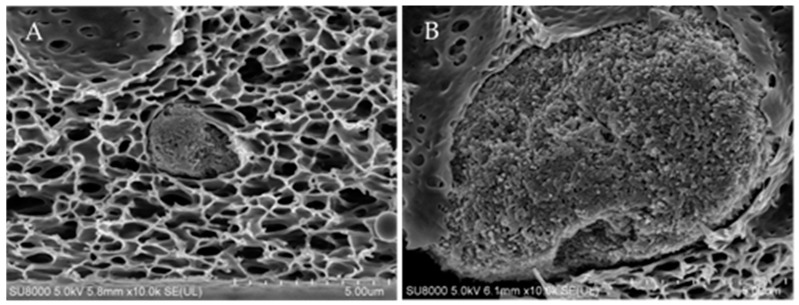
SEM images of HNTs agglomerates in 1%HNT (**A**) and 4%HNT (**B**) membranes.

In case of the HNTs-modified membranes the presence of the nanofiller was confirmed based on SEM-BSE images (Figure 4, left column). The number and diameters of HNTs clusters visible in the membranes cross sections were higher in case of higher HNTs concentrations. For 0.5%HNT and 1%HNT membranes the halloysite nanotubes were hardly to be found, while in case of the membranes containing HNTs loadings above 2 wt % the clusters were clearly visible. Moreover, in general much smaller agglomerates (Figure 5A) were formed in membranes containing low amount of halloysite compared to those with high HNTs content (Figure 5B). The largest clusters were visible in membranes containing 3 and 4 wt % of halloysite. Moreover, during the analysis of the 4%HNT membrane mainly large agglomerates similar to that shown in Figure 5B were observed. Nonetheless, no dependence between the HNTs content and the location and density of the clusters was found, their distribution was random.

Furthermore, when small HNTs agglomerates were formed, the structure of the membranes was almost not affected (Figure 4B). On the opposite, the presence of large agglomerates led to significant changes in the size and shape of the finger-like pores. The cross sections of the 1% HNT (Figure 4C) and 2%HNT (Figure 4D) membranes revealed the formation of distorted elongated pores ended with a HNTs cluster. The greatest effect on the shape of pores was observed for the 4%HNT membrane (Figure 4F).

**Figure 6:**
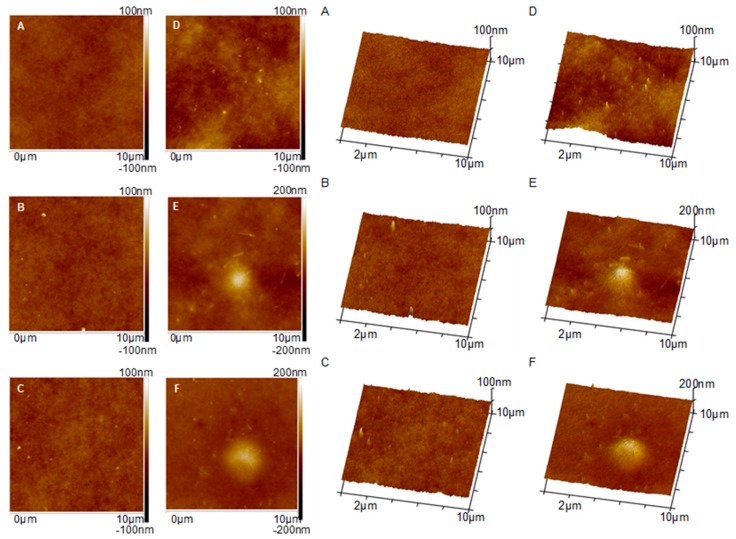
AFM images of the surface of (**A**) 0%HNT, (**B**) 0,5%HNT, (**C**) 1%HNT, (**D**) 2%HNT, (**E**) 3%HNT, (**F**) 4%HNT membranes.

Figure 6 shows AFM images of the membranes surface visualized in 2D (left column) and 3D (right column) modes. In case of the membranes containing the lowest halloysite amount (0.5%HNT, 1%HNT and 2%HNT) some single, well dispersed nanoparticles and their aggregates with diameters in the range of ca. 30–270 nm can be observed (Figure 6B–D). Membranes modified with a higher amount of the nanofiller (3%HNT and 4%HNT) contained, except for small aggregates of nanoparticles, also larger agglomerates (Figure 6E,F). On the surface of the membrane containing 3 wt % of halloysite nanotubes, the agglomerate with a diameter of about 2 µm is visible (Figure 6E), while on the surface of the membrane modified with 4 wt % of HNTs the diameter of the agglomerate exceeds 3 µm. The results indicate that a good dispersion of halloysite on the surface of the membranes can be obtained only at lower HNTs loadings, up to 2 wt %. Further increase of the amount of nanoparticles resulted in the formation of large agglomerates. 

According to Buruga el al. [22], halloysite nanotubes have very few functional groups on their exterior surface, which promotes their good dispersion in polymer matrix. However, the results obtained in the present study revealed that halloysite nanotubes form agglomerates, especially at higher content, what was confirmed using AFM (Figure 6) and SEM analyses (Figure 4). The presence of these agglomerates can negatively affect the surface properties of the membranes due to uneven distribution of HNTs.

**Figure 7:**
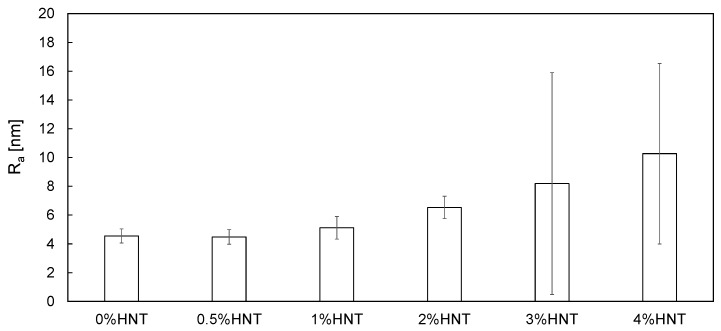
The influence of HNTs content on surface roughness (*R*_a_) of membranes.

Based on AFM images (Figure 6) the surface roughness (*R*_a_) of the membranes was estimated (Figure 7).

**Figure 8:**
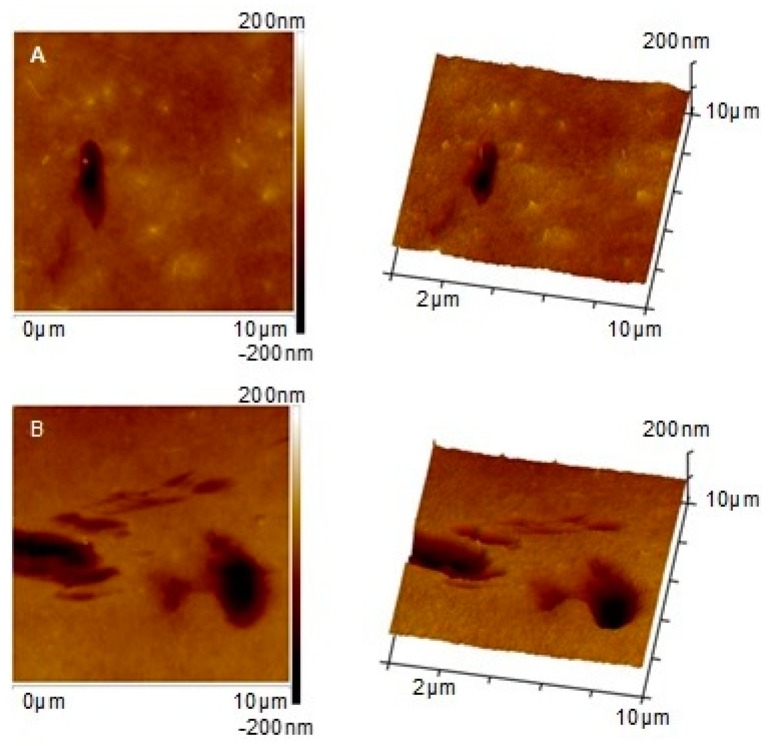
AFM images of the surface of 3%HNT (**A**) and 4%HNT (**B**) membranes presenting the imperfections.

The analysis (Figure 7) revealed an increase of roughness with increasing HNTs content. The R_a_ value of the membrane containing 0.5 wt % of HNTs was relatively low (4.50(0.51) nm) and similar to that calculated for the unmodified membrane (4.55(0.49) nm). This is well reflected by the AFM image of the 0.5%HNT membrane surface (Figure 6B) where the halloysite nanotubes are very rarely found. In case of membranes modified with higher content of the nanofiller, the R_a_ increased gradually, reaching 5.12(0.78) nm for 1%HNT and 10.26(6.27) nm for 4%HNT. That growth of roughness is related to the introduction of larger amount of nanomaterial and higher probability of formation of agglomerates by HNTs, which can be observed in Figure 6E,F. The increase of roughness of 2%HNT membrane (Figure 7) in comparison to the membranes containing lower loading of the nanofiller was mainly associated with the presence of higher amount of halloysite nanotubes on the surface of the membrane (Figure 6D). However, in case of 3%HNT and 4%HNT membranes some other factor contributed to the calculated R_a_ values. Except from large HNTs aggregates, some imperfections resembling large pores were present. An example of the surface of 3%HNT and 4%HNTmembranes containing such imperfections is shown in Figure 8. The analysis of Figure 8B revealed the occurrence of large HNTs agglomerate with a diameter of 1.7 µm and, in its proximity, a “hole” with the dimensions similar to the cluster of halloysite nanotubes. Appearance of such large pores can be explained by the removal of nanoparticles from the polymer film at the stage of membrane preparation (film casting or gelation). These imperfections are well represented by the SD values of R_a_. As can be seen in Figure 7, in case of the membranes containing low amount of HNTs the error bars were relatively small, while at higher nanofiller content, especially 3 and 4 wt %, they were significantly larger, showing the non-uniformity of the membranes surface.

**Figure 9:**
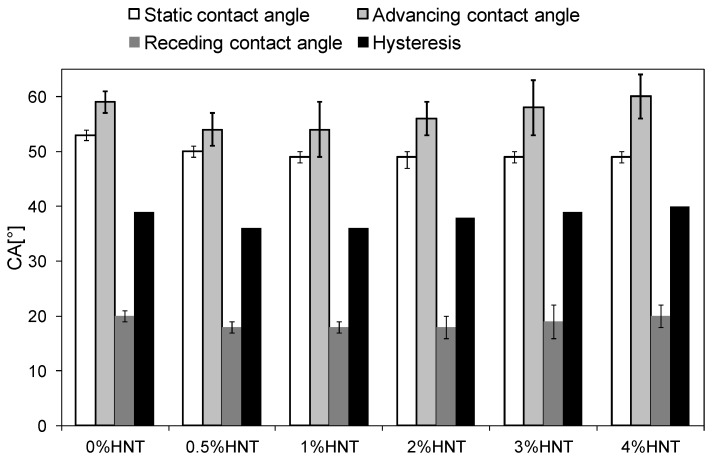
Static, advancing and receding contact angles and contact angle hysteresis of the unmodified and HNTs-modified membranes.

The addition of HNTs influenced also the hydrophilicity of the obtained membranes. The static water contact angles (SCA) were in the range of 49 to 53° being the highest for the unmodified membrane (Figure 9). The positive influence of HNTs on hydrophilicity was also observed by Buruga et al. [22] in case of polystyrene membranes. The decrease of SCA values of the mixed-matrix membranes was attributed to the presence of –OH groups in their structure resulting from the introduction of the filler. As can be seen in Figure 9, SCA values decreased with the increase of halloysite nanotubes content up to 1 wt %. Application of higher HNTs loadings did not contribute to further increase of membranes hydrophilicity, which is probably related to the roughness of the membranes obtained. The dependence of wetting characteristic of a solid on the surface roughness was already discussed by Wenzel [42]. The influence of roughness on the nature of the surface wetting was also recently reported by Kubiak and others [43]. Sotto et al. [44] contributed the changes of SCA values of PES membranes modified with TiO_2_ to agglomeration of the nanoparticles. They reported that for the same NPs amount the membranes containing larger TiO_2_ agglomerates exhibited lower hydrophilicity. In view of the above it can be concluded that the similar values of SCA of the membranes containing ≥2 wt % of HNTs (Figure 9), resulted from the agglomeration of the nanoparticles affecting the roughness of the membranes; however, since concomitantly the content of hydrophilic halloysite was increased from 2 to 4 wt % the SCA value eventually remained constant.

Aside from the SCA measurements, the surface of the membranes was furtherly analyzed by assessing their advancing and receding contact angles. ACA is a measure of the overall hydrophobic character of the surface, while RCA reflects its relative hydrophilic properties [45]. The obtained advancing and receding contact angles are shown in Figure 9, along with the hysteresis calculated as the difference between the respective ACA and RCA values. Although the differences between the results obtained for various membranes were not very significant, some relations among HNTs content and CA values can be found. The ACA of the membranes containing from 0.5 to 3 wt % of HNTs was lower than that of unmodified membrane, with the most noticeable decrease observed for 0.5%HNT and 1%HNT samples. In case of the membranes modified with 2–4 wt % of HNTs the ACA values were gradually increasing with the addition of the modifier, compared to that measured for lower content of the nanofiller. The advanced contact angle of 4%HNT reached 60°, being even slightly higher than that of 0%HNT (59°). Similar course of changes was observed in case of RCA. The receding contact angle of the unmodified membrane was 20°. In the case of the modified membranes containing 0.5–2 wt % of halloysite, the RCA remained stabilized at 18°. Further increase of HNTs content resulted in a slight increase of RCA to the value determined for the unmodified membrane. The hysteresis increased from 36° for 0.5%HNT to 40° for 4%HNT, indicating the increase of heterogeneity of the membranes surface [45,46,47]. This trend of hysteresis changes is well reflected by the membranes surface roughness (Figure 6 and Figure 7) and confirms the conclusions drawn on a basis of SCA values. A lower hysteresis determined for 0.5%HNT and 1%HNT compared to the unmodified membrane was resulting from the hydrophilic character of the incorporated HNTs. This statement is supported by the similar R_a_ values (Figure 7) of the mentioned three membranes. The increase of hysteresis with increasing content of the nanofiller reflects the increasing roughness of the membranes surface. As a result, no further improvement of membranes hydrophilicity is observed. A similar phenomenon was observed by Celik et al. [48] during their investigations on multi-walled carbon nanotubes (MWCNTs) blended polyethersulfone membranes. They found that increasing the MWCNTs content above 2% did not result in further enhancement of the hydrophilicity of membranes. Such behavior can be explained in terms of Cassie–Baxter model [49] as was proposed by Grosso et al. [50]. According to the model the increase of surface roughness of the hydrophilic material results in the reduction of its hydrophilic properties.

The presence of HNTs should affect the surface charge of the membranes. The measured isoelectric point of the applied HNTs is 1.9, which means that at pH above this value the surface of the nanotubes is negatively charged. As a result, one can expect a more negative surface of the modified membranes compared to the neat one. This supposition was confirmed by the zeta potential analysis of the membranes (Table 1).

The isoelectric point of the unmodified membrane (2.8) was higher than that of the HNTs-modified membranes, except for the membrane containing 4 wt % of the nanofiller (2.9). The lowest pH(I) was found in case of the 0.5%HNT membrane and the value of this parameter was increasing with increasing HNTs content. Comparing the obtained results with surface roughness (Figure 7) of the modified membranes it can be found that the increase in pH(I) correlates with the increase in R_a_. Schnitzer et al. [51] reported the influence of surface roughness (in the micrometers range) on the zeta potential of polyester plates determined by the streaming potential method. However, the authors did not find any effect of the roughness on the isoelectric point of the material. On the opposite, Borghi et al. [52] observed a decrease in the pH(I) value with an increase in the roughness of nanostructured TiO_2_ thin films. A more complex analysis was presented by Lützenkirchenet al. [53] in their studies on various factors influencing pH(I) of sapphire-c (α-alumina). The authors reported that atomically smooth surfaces are characterized by lower pH(I) values, whereas rougher surfaces (exhibiting roughness on the order of nanometers) show higher pH(I). However, in case of very rough sapphire-c surfaces (roughness on the order of micrometers) the isoelectric point decreased compared to the reference sample with nanometer-scale roughness. Furthermore, they found that pH(I) determined by streaming potential method and static colloid adhesion measurement differed, which was explained with reference to the role of hydrodynamics in streaming potential experiments [53]. All the above show a very complex nature of the isoelectric point determination in case of rough surfaces, which becomes even much more complicated when nanocomposites such as mixed-matrix membranes are considered. Nonetheless, the results obtained in this work revealed that increasing roughness of HNTs-modified membranes was accompanied by increasing pH(I) values.

### 3.3. Permeability of the Membranes

**Figure 10:**
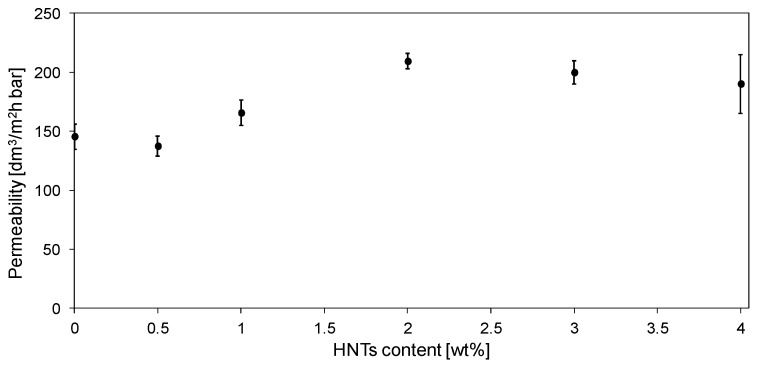
The influence of the HNTs loading on pure water flux through PES membranes.

The influence of HNTs content on membranes permeability is summarized in Figure 10.

No significant influence of the introduction of 0.5 wt % of halloysite nanotubes into the membrane matrix on the permeability was found, despite the increase in total porosity and hydrophilicity of the 0.5%HNT membrane compared to the unmodified one (Figure 9). Based on the SEM image (Figure 4B), it can be seen that part of the introduced filler formed agglomerates in the interior of the membrane matrix, thus influencing the total porosity. However, considering that the amount of halloysite nanotubes in 0.5%HNT membrane was low, their presence did not affect significantly the porosity of the separation layer, opposite to the membranes with higher content of HNTs, as was observed in the AFM images (Figure 6). Therefore, the pure water flux was not significantly influenced in case of the lowest filler content. The highest enhancement of permeability (by ca. 44% compared to the unmodified membrane) was achieved after introduction of 2 wt % of HNTs (Figure 10). Similar results were obtained by Zhang et al. [10] who reported an increase of pure water flux due to HNTs addition up to 3 wt % in PES membranes prepared using DMAc as a solvent. Ghanbari et al. [19] explained the increase in the permeate flux by tubular structure of halloysite. Based on AFM images (Figure 6) the improvement of permeability can also be explained by additional pores created by HNTs themselves. However, from Figure 10 it can be found that an increase of HNTs loading up to 3 and 4 wt % did not contribute to pure water flux improvement compared to 2%HNT membrane. The obtained results can be related to the formation of aggregates and agglomerates blocking the surface of the membrane, as was observed in AFM images (Figure 6E,F).

### 3.4. Membranes Fouling by BSA

**Figure 11:**
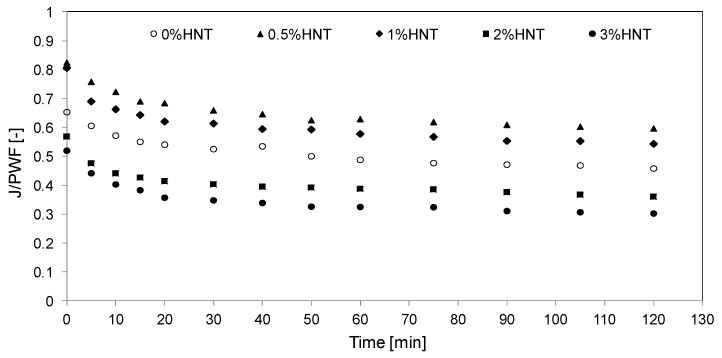
The effect of HNTs content on BSA fouling of the PES membranes. Initial BSA concentration: 1 g/dm^3^; TMP = 2 bar.

The antifouling properties of the membranes modified with 0.5–3 wt % of HNTs were determined using bovine serum albumin as a model foulant (Figure 11). The 4%HNT membrane was not tested due to (i) numerous imperfections in the membrane skin layer (Figure 8) and (ii) the decreasing resistance to fouling of 2%HNT and 3%HNT membranes compared to the unmodified one. During the BSA ultrafiltration process, the decrease of permeate flux through the unmodified membrane in comparison to PWF reached 54% after 2 h of the experiment, whereas for the 0.5%HNT and 1%HNT membranes the permeate flux lowered for 40% and 46%, respectively. The introduction of a larger amount of the filler resulted in a deterioration of antifouling properties and a severe decrease of permeate flux by 64% in case of 2%HNT and 70% for 3%HNT membranes (Figure 11).

Duan et al. [35] observed the best antifouling properties for a membrane containing as much as 3 wt % of HNTs modified by *N*-halamine. During the BSA ultrafiltration process, a 44% decrease in permeate flux was observed by the authors for the unmodified PES membrane, while 37.5% drop occurred after introduction of the filler. This shows only 6.5 percentage point (p.p.) improvement. Application of 3 wt % of AgNPs-rGO-HNTs composite presented by Zhao et al. [27] resulted in ca. 12 p.p. increase of permeate flux during BSA ultrafiltration compared to unmodified PES membrane. Considering the 14 p.p. increase in permeate flux observed for 0.5%HNT and the 8 p.p. increase in case of 1%HNT membranes (Figure 11), it can be concluded that the neat HNTs introduced into the polymer matrix can improve antifouling performance of the membranes in a similar manner as the modified nanotubes. It should also be mentioned,= that in both the above discussed papers [27,35] the amount of the nanofiller was high (3 wt %). The results presented in Figure 8 show that such a content of HNTs may result in formation of imperfections on the membrane surface. Therefore, application of such high loading of the NPs should be carefully considered. The results obtained in the present study (Figure 11) revealed that a significant fouling mitigation in case of neat HNTs can be obtained at low NPs loadings (0.5–1 wt %), in which case the membrane surface was free of imperfections.

**Figure 12:**
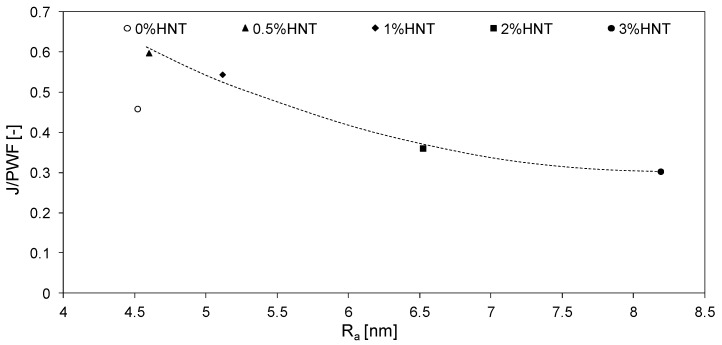
Dependence of permeate flux decline during UF of BSA solution on surface roughness (R_a_) of the membranes. The line is added to guide the eye.

The antifouling properties of the HNTs-modified membranes can be explained in terms of the increase in hydrophilicity and the more negative charge of the membrane surface causing the repulsion of the negatively charged BSA [28]. The zeta potential of the mixed matrix membranes obtained in the present study, measured at pH corresponding to pH of the BSA solution (6.85), was similar for all HNTs loadings and ranged from −36 to −38 mV. The zeta potential of the unmodified membrane was significantly higher and amounted to −27 mV. The more negative zeta potential of the modified membranes resulted from the presence of halloysite nanotubes on their surface (Figure 6). In general, the negative charge accumulated on the surface of the membranes should improve their antifouling properties due to a stronger repulsion of the negatively charged BSA molecules. However, considering the drastic deterioration of the permeate flux in case of membranes containing more than 2 wt % (Figure 11) some other factors affecting their antifouling properties should be taken into account. It was proposed that the results presented in Figure 11 can be explained in terms of membrane roughness (R_a_). Figure 12 shows the dependence of the permeate flux decrease on the surface roughness of the obtained membranes.

The membranes modified with lower filler amount were less prone to BSA fouling than those modified with high HNTs loading. A significant decrease in fouling resistance observed for 2%HNT and 3%HNT membranes was associated with increased roughness and, as in the case of the 3%HNT membrane, occurrence of imperfections (holes) in the membrane skin (Figure 8). The presence of larger pores and holes, similar to the increased roughness, was conducive to the deposition of BSA on the surface of the membrane, thereby reducing the efficiency of the ultrafiltration process. Liu et al. [54] during their investigations on composite membranes made of PES, halloysite and modified halloysite found that membranes with a relatively smooth surface were more resistant to fouling. Hobbs et al. [55] also observed that an increase in roughness caused a deterioration of antifouling properties, which is consistent with the results presented in Figure 11 and Figure 12.

No significant influence of the modification on the retention of BSA was observed. The rejection of the model compound by the unmodified membrane and the membranes containing the lowest nanofiller amount (i.e., 0.5 and 1 wt %) was 99.4(0.1)%, and changed only slightly when the HNTs loading increased up to 2 wt % (99.2(0.2)%) and 3 wt % (99.1(0.3)%).

## 4. Conclusions

The influence of HNTs on physicochemical, transport and antifouling properties of mixed-matrix PES membranes was presented and discussed. The introduction of HNTs had a positive effect on membrane hydrophilicity due to the presence of –OH groups in the nanofiller. However, no difference between static water contact angles measured for membranes containing more than 1 wt % of HNTs was observed. That phenomenon, together with the increasing contact angle hysteresis at higher HNTs loading, was explained in terms of membrane roughness. Based on SEM and AFM analysis of the membranes structure it was proved that much smaller HNTs agglomerates were formed in case of low halloysite content compared to high amount of the filler. It was concluded that a good dispersion of HNTs on the membrane surface can be obtained only at HNTs loadings below 2 wt %.

An increase of membrane roughness with increasing HNTs content was observed. In case of 3%HNT and 4%HNT membranes the R_a_ value was affected by some imperfections resembling large pores present in the skin layer. Their appearance was explained by the removal of HNTs from the polymer film at the stage of membrane preparation.

The presence of HNTs influenced the surface charge of the membranes. It was found that an increase of the roughness of HNTs-modified membranes was accompanied by an increase of isoelectric point values.

An improvement of membrane permeability with increasing HNTs loading up to 2 wt % was observed. However, addition of higher amount of HNTs did not contribute to further increase of pure water flux what was related to the formation of HNTs agglomerates in the membrane separation layer.

A relation between severity of membrane fouling and surface roughness was proved. In general, membranes with a lower filler content and exhibiting a smoother surface were less prone to BSA fouling than those modified with high HNTs amount and characterized by a rough surface.

## Figures and Tables

**Table 1:** Isoelectric point of the neat and HNTs-modified PES membranes.

Membrane	pH(I)
0%HNT	2.8 (0.1)
0.5%HNT	2.2 (0.0)
1%HNT	2.4 (0.1)
2%HNT	2.5 (0.0)
3%HNT	2.5 (0.1)
4%HNT	2.9 (0.1)
